# Supplementary material for: Modeling glioblastoma heterogeneity as a dynamic network of cell states
Source: Mol Syst Biol. 2021 Sep 16;17(9):e10105. doi: 10.15252/msb.202010105 (PMC8444284; doi:10.15252/msb.202010105)
Supplement: Supplementary file 5 — Source Data for Figure 3 [file MSB-17-e10105-s001.zip › Figure3A_sourcedata/GSEA_3065/hallmarks_state3.GseaPreranked.1623416274744/gsea_report_for_na_pos_1623416274744.html]

Report for na\_pos 1623416274744 [GSEA]

| GS  follow link to MSigDB | GS DETAILS | SIZE | ES | NES | NOM p-val | FDR q-val | FWER p-val | RANK AT MAX | LEADING EDGE || 1 | HALLMARK\_EPITHELIAL\_MESENCHYMAL\_TRANSITION | Details ... | 117 | 0.55 | 2.21 | 0.000 | 0.000 | 0.000 | 1291 | tags=50%, list=15%, signal=58% |
| 2 | HALLMARK\_ANGIOGENESIS | Details ... | 20 | 0.76 | 2.15 | 0.000 | 0.000 | 0.000 | 1358 | tags=70%, list=16%, signal=83% |
| 3 | HALLMARK\_MITOTIC\_SPINDLE | Details ... | 173 | 0.48 | 2.05 | 0.000 | 0.000 | 0.000 | 1338 | tags=31%, list=15%, signal=36% |
| 4 | HALLMARK\_CHOLESTEROL\_HOMEOSTASIS | Details ... | 60 | 0.51 | 1.85 | 0.000 | 0.001 | 0.003 | 1011 | tags=40%, list=12%, signal=45% |
| 5 | HALLMARK\_MTORC1\_SIGNALING | Details ... | 169 | 0.39 | 1.65 | 0.000 | 0.020 | 0.049 | 1050 | tags=26%, list=12%, signal=29% |
| 6 | HALLMARK\_G2M\_CHECKPOINT | Details ... | 177 | 0.39 | 1.65 | 0.000 | 0.016 | 0.049 | 1394 | tags=29%, list=16%, signal=34% |
| 7 | HALLMARK\_INFLAMMATORY\_RESPONSE | Details ... | 68 | 0.41 | 1.49 | 0.016 | 0.068 | 0.232 | 1483 | tags=31%, list=17%, signal=37% |
| 8 | HALLMARK\_ANDROGEN\_RESPONSE | Details ... | 68 | 0.41 | 1.47 | 0.019 | 0.074 | 0.283 | 450 | tags=22%, list=5%, signal=23% |
| 9 | HALLMARK\_PROTEIN\_SECRETION | Details ... | 87 | 0.38 | 1.44 | 0.014 | 0.080 | 0.332 | 816 | tags=24%, list=9%, signal=26% |
| 10 | HALLMARK\_UV\_RESPONSE\_DN | Details ... | 104 | 0.36 | 1.42 | 0.009 | 0.090 | 0.391 | 2037 | tags=32%, list=23%, signal=41% |
| 11 | HALLMARK\_HEDGEHOG\_SIGNALING | Details ... | 23 | 0.49 | 1.40 | 0.084 | 0.090 | 0.416 | 1022 | tags=30%, list=12%, signal=34% |
| 12 | HALLMARK\_UNFOLDED\_PROTEIN\_RESPONSE | Details ... | 98 | 0.34 | 1.32 | 0.044 | 0.159 | 0.641 | 1082 | tags=20%, list=12%, signal=23% |
| 13 | HALLMARK\_E2F\_TARGETS | Details ... | 185 | 0.31 | 1.32 | 0.028 | 0.148 | 0.644 | 1426 | tags=25%, list=16%, signal=29% |
| 14 | HALLMARK\_COAGULATION | Details ... | 54 | 0.37 | 1.32 | 0.086 | 0.138 | 0.647 | 988 | tags=30%, list=11%, signal=33% |
| 15 | HALLMARK\_ESTROGEN\_RESPONSE\_EARLY | Details ... | 99 | 0.34 | 1.31 | 0.057 | 0.135 | 0.666 | 2247 | tags=39%, list=26%, signal=52% |
| 16 | HALLMARK\_WNT\_BETA\_CATENIN\_SIGNALING | Details ... | 27 | 0.44 | 1.31 | 0.104 | 0.133 | 0.691 | 1914 | tags=26%, list=22%, signal=33% |
| 17 | HALLMARK\_APICAL\_SURFACE | Details ... | 20 | 0.47 | 1.30 | 0.151 | 0.128 | 0.696 | 1232 | tags=30%, list=14%, signal=35% |
| 18 | HALLMARK\_MYOGENESIS | Details ... | 95 | 0.33 | 1.28 | 0.050 | 0.146 | 0.764 | 1139 | tags=22%, list=13%, signal=25% |
| 19 | HALLMARK\_COMPLEMENT | Details ... | 93 | 0.33 | 1.28 | 0.052 | 0.139 | 0.765 | 398 | tags=16%, list=5%, signal=17% |
| 20 | HALLMARK\_IL2\_STAT5\_SIGNALING | Details ... | 101 | 0.32 | 1.26 | 0.053 | 0.147 | 0.808 | 1036 | tags=23%, list=12%, signal=26% |
| 21 | HALLMARK\_APICAL\_JUNCTION |  | 113 | 0.30 | 1.19 | 0.114 | 0.235 | 0.938 | 1079 | tags=23%, list=12%, signal=26% |
| 22 | HALLMARK\_IL6\_JAK\_STAT3\_SIGNALING |  | 32 | 0.37 | 1.18 | 0.218 | 0.245 | 0.954 | 1222 | tags=25%, list=14%, signal=29% |
| 23 | HALLMARK\_ALLOGRAFT\_REJECTION |  | 59 | 0.31 | 1.09 | 0.276 | 0.394 | 0.997 | 1309 | tags=31%, list=15%, signal=36% |
| 24 | HALLMARK\_ESTROGEN\_RESPONSE\_LATE |  | 99 | 0.27 | 1.06 | 0.315 | 0.441 | 0.998 | 1036 | tags=18%, list=12%, signal=20% |
| 25 | HALLMARK\_APOPTOSIS |  | 107 | 0.26 | 1.02 | 0.406 | 0.544 | 1.000 | 1060 | tags=21%, list=12%, signal=23% |
| 26 | HALLMARK\_INTERFERON\_ALPHA\_RESPONSE |  | 55 | 0.28 | 0.99 | 0.474 | 0.597 | 1.000 | 1046 | tags=13%, list=12%, signal=14% |
| 27 | HALLMARK\_KRAS\_SIGNALING\_UP |  | 83 | 0.26 | 0.98 | 0.511 | 0.604 | 1.000 | 1738 | tags=25%, list=20%, signal=31% |
| 28 | HALLMARK\_UV\_RESPONSE\_UP |  | 111 | 0.24 | 0.94 | 0.595 | 0.686 | 1.000 | 697 | tags=12%, list=8%, signal=13% |
| 29 | HALLMARK\_SPERMATOGENESIS |  | 54 | 0.26 | 0.93 | 0.585 | 0.706 | 1.000 | 2012 | tags=26%, list=23%, signal=33% |
| 30 | HALLMARK\_GLYCOLYSIS |  | 139 | 0.23 | 0.93 | 0.660 | 0.687 | 1.000 | 1937 | tags=29%, list=22%, signal=37% |
| 31 | HALLMARK\_NOTCH\_SIGNALING |  | 22 | 0.31 | 0.90 | 0.601 | 0.735 | 1.000 | 1990 | tags=41%, list=23%, signal=53% |
| 32 | HALLMARK\_XENOBIOTIC\_METABOLISM |  | 93 | 0.23 | 0.89 | 0.683 | 0.724 | 1.000 | 804 | tags=14%, list=9%, signal=15% |
| 33 | HALLMARK\_BILE\_ACID\_METABOLISM |  | 51 | 0.22 | 0.77 | 0.882 | 0.920 | 1.000 | 2399 | tags=37%, list=27%, signal=51% |
Table: Gene sets enriched in phenotype **na **[plain text format]****
